# Supplementary material for: Nigeria healthcare worker SARS-CoV-2 serology study: Results from a prospective, longitudinal cohort
Source: PLOS Glob Public Health. 2023 Jan 17;3(1):e0000549. doi: 10.1371/journal.pgph.0000549 (PMC10022168; doi:10.1371/journal.pgph.0000549)
Supplement: S1 Table — (DOCX) [file pgph.0000549.s001.docx]

**S1 Table.** Baseline study participants’ symptoms to SARS-CoV-2 by age group.

| Baseline Characteristic | Total respondents  N (525) | Age <40 years  (N=278) | Age > 40 years  (N=247) | *P-*value |
| --- | --- | --- | --- | --- |
| Any symptoms, No. (%) | 509 | 32 (11.8) | 32 (13.4) | 0.58 |
| Fever, No. (%) | 64 | 20 (62.5) | 22 (68.8) | 0.60 |
| Shortness of breath, No. (%) | 63 | 1 (3.2) | 0 (0.0) | 0.31 |
| Chest congestion, No. (%) | 63 | 1 (3.2) | 0 (0.0) | 0.31 |
| Chest tightness, No. (%) | 63 | 0 (0.0) | 2 (6.3) | 0.16 |
| Dry cough, No. (%) | 61 | 1 (3.4) | 1 (3.1) | 0.94 |
| Wet/loose cough, No. (%) | 61 | 1 (3.4) | 3 (9.4) | 0.35 |
| Body aches or pains, No. (%) | 61 | 16 (55.2) | 24 (75.0) | 0.10 |
| Chills or shivering, No. (%) | 62 | 8 (26.7) | 4 (12.5) | 0.16 |
| Sore throat, No. (%) | 62 | 1 (3.3) | 2 (6.3) | 0.59 |
| Congested or runny nose, No. (%) | 62 | 6 (20.0) | 5 (15.6) | 0.65 |
| Diarrhea, No. (%) | 62 | 3 (10.0) | 1 (3.1) | 0.27 |
| Weak or tired, No. (%) | 61 | 20 (66.7) | 16 (51.6) | 0.23 |
| Loss of smell, No. (%) | 61 | 0 (0.0) | 1 (3.1) | 0.34 |
| Loss of taste, No. (%) | 62 | 4 (13.3) | 2 (6.3) | 0.35 |
| Loss of appetite, No. (%) | 62 | 11 (36.7) | 9 (28.1) | 0.47 |
| Vomiting, No. (%) | 61 | 1 (3.3) | 1 (3.2) | 0.98 |
| Nausea, No. (%) | 61 | 1 (3.4) | 4 (12.5) | 0.20 |
| Headache, No. (%) | 62 | 25 (83.3) | 19 (59.4) | 0.04 |
| Abdominal pain, No. (%) | 62 | 4 (13.3) | 2 (6.3) | 0.35 |

Excluded: 16 participants were missing data on questions about symptoms: 15 participants believed they had COVID-19 because they were ill but either tested negative or did not test, and 1 was missing data on symptoms.
