# Supplementary material for: Nigeria healthcare worker SARS-CoV-2 serology study: Results from a prospective, longitudinal cohort
Source: PLOS Glob Public Health. 2023 Jan 17;3(1):e0000549. doi: 10.1371/journal.pgph.0000549 (PMC10022168; doi:10.1371/journal.pgph.0000549)
Supplement: S2 Table — (DOCX) [file pgph.0000549.s002.docx]

**S2 Table.** COVID-19 symptoms, diagnostic testing, diagnosis, and hospitalizations among participants at baseline, 3-month, and 6- month follow-up.

|  | Baseline  (n=525) | 3-Month Follow-up  (n=503) | 6-Month Follow-up  (n=491) |
| --- | --- | --- | --- |
| Symptoms of COVID-19 | 64 (12.2%) | 33 (6.6%) | 37 (7.5%) |
| Diagnostic testing for COVID-19 | 61 (11.6%) | 5 (1.0%) | 24 (4.9%) |
| COVID diagnosis | 4 (0.7%) | 1 (0.1%) | 8 (1.6%) |
| Hospitalization | 0 | 0 | 1 (0.2%) |
| ICU stay | 0 | 0 | 0 |

*All participants included in the analysis irrespective of seropositivity or mishandled, missing, or insufficient samples, which explains the difference between the number and proportion of participants with symptoms at baseline in this table compared with Supplemental Table 3.

ICU=intensive care unit
