# Supplementary figures and images for: Nigeria healthcare worker SARS-CoV-2 serology study: Results from a prospective, longitudinal cohort
Source: PLOS Glob Public Health. 2023 Jan 17;3(1):e0000549. doi: 10.1371/journal.pgph.0000549 (PMC10022168; doi:10.1371/journal.pgph.0000549)

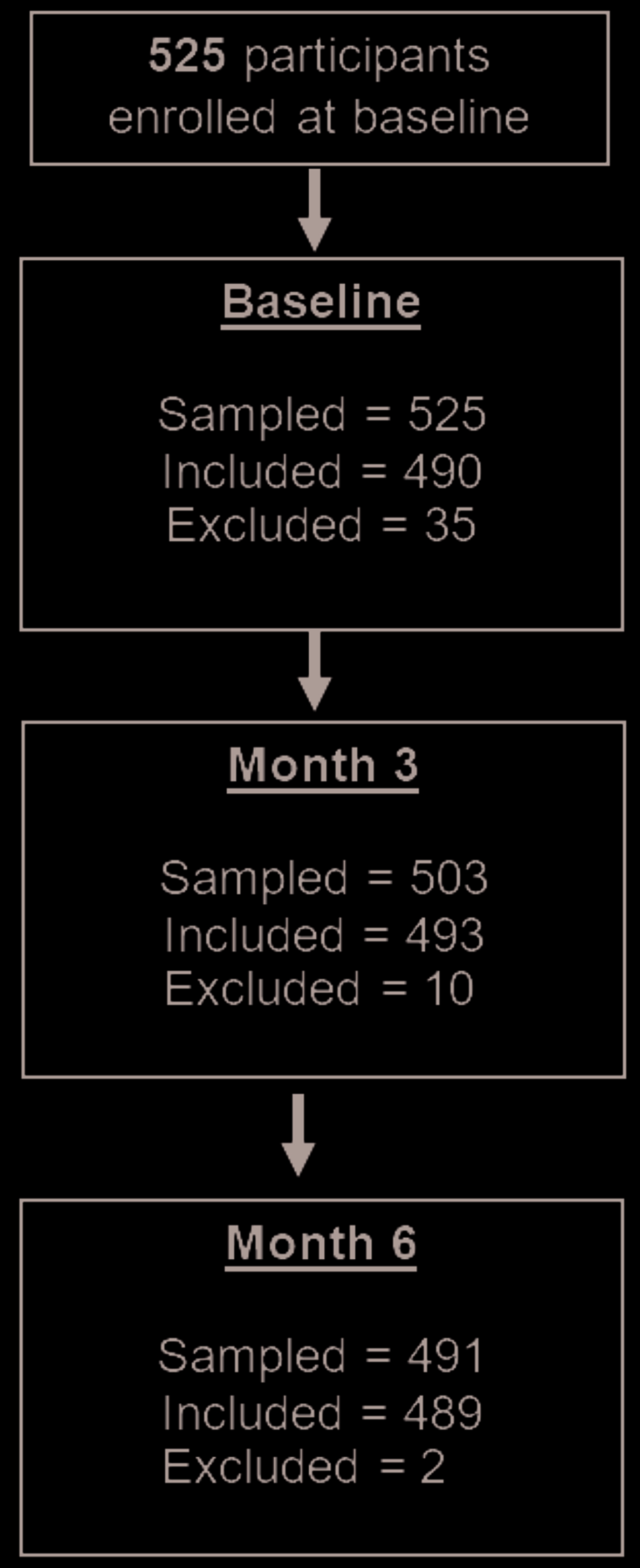

Supplement: S1 Fig — (TIF) [file pgph.0000549.s003.tif]

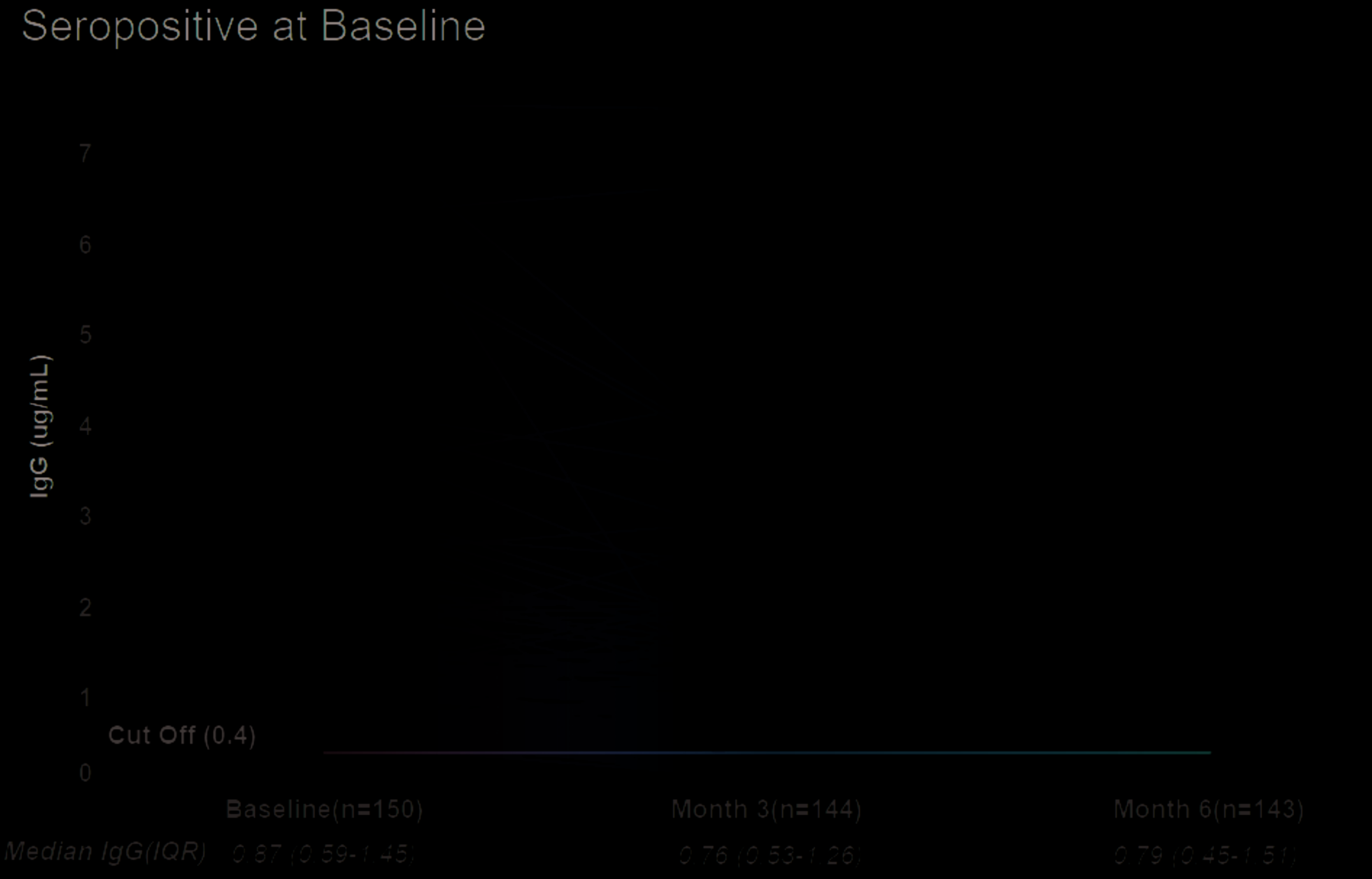

Supplement: S2 Fig — *Ptrend = 0.08. (TIF) [file pgph.0000549.s004.tif]

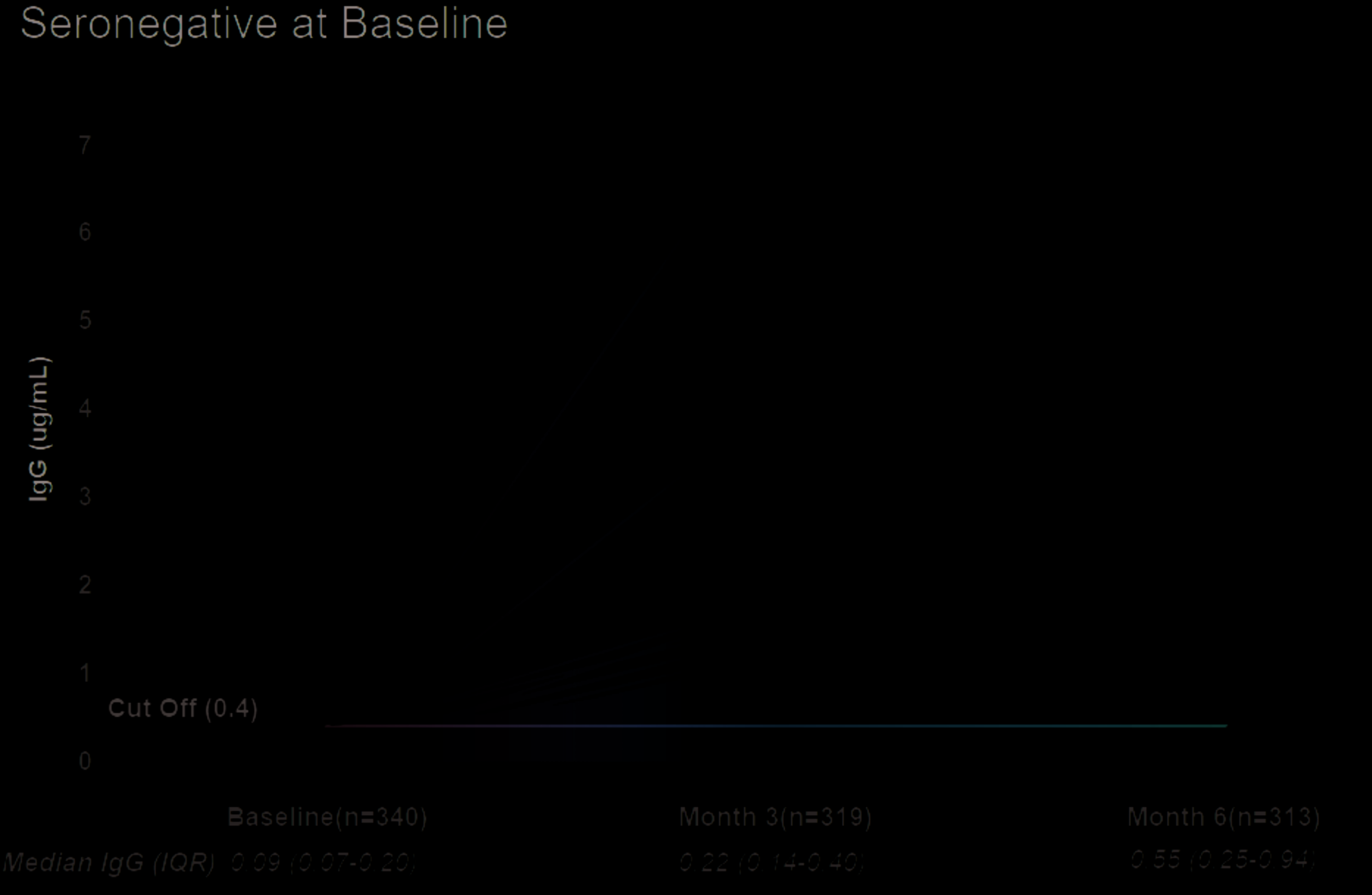

Supplement: S3 Fig — *Ptrend = < .0001. (TIF) [file pgph.0000549.s005.tif]

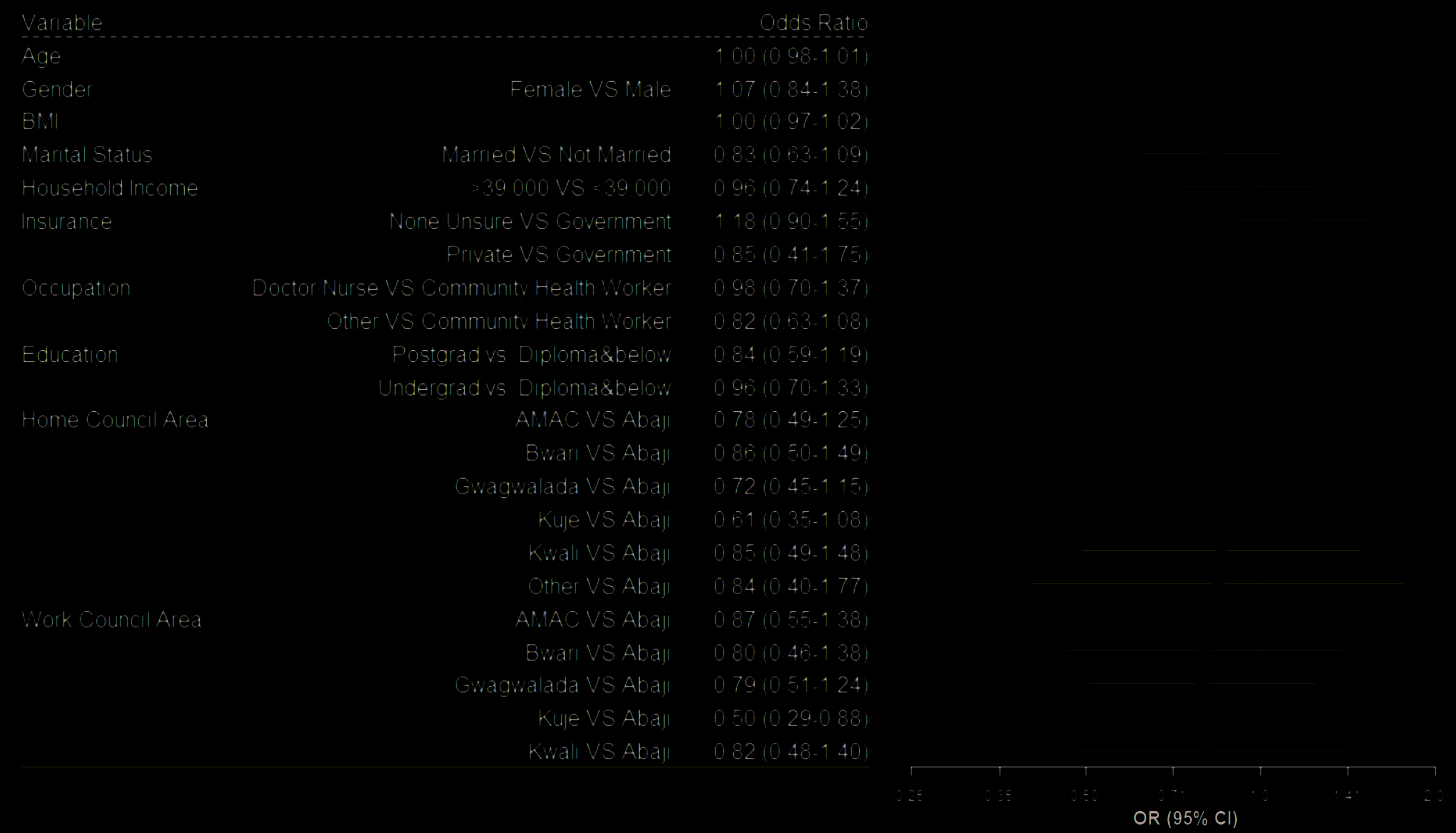

Supplement: S4 Fig — (TIF) [file pgph.0000549.s006.tif]

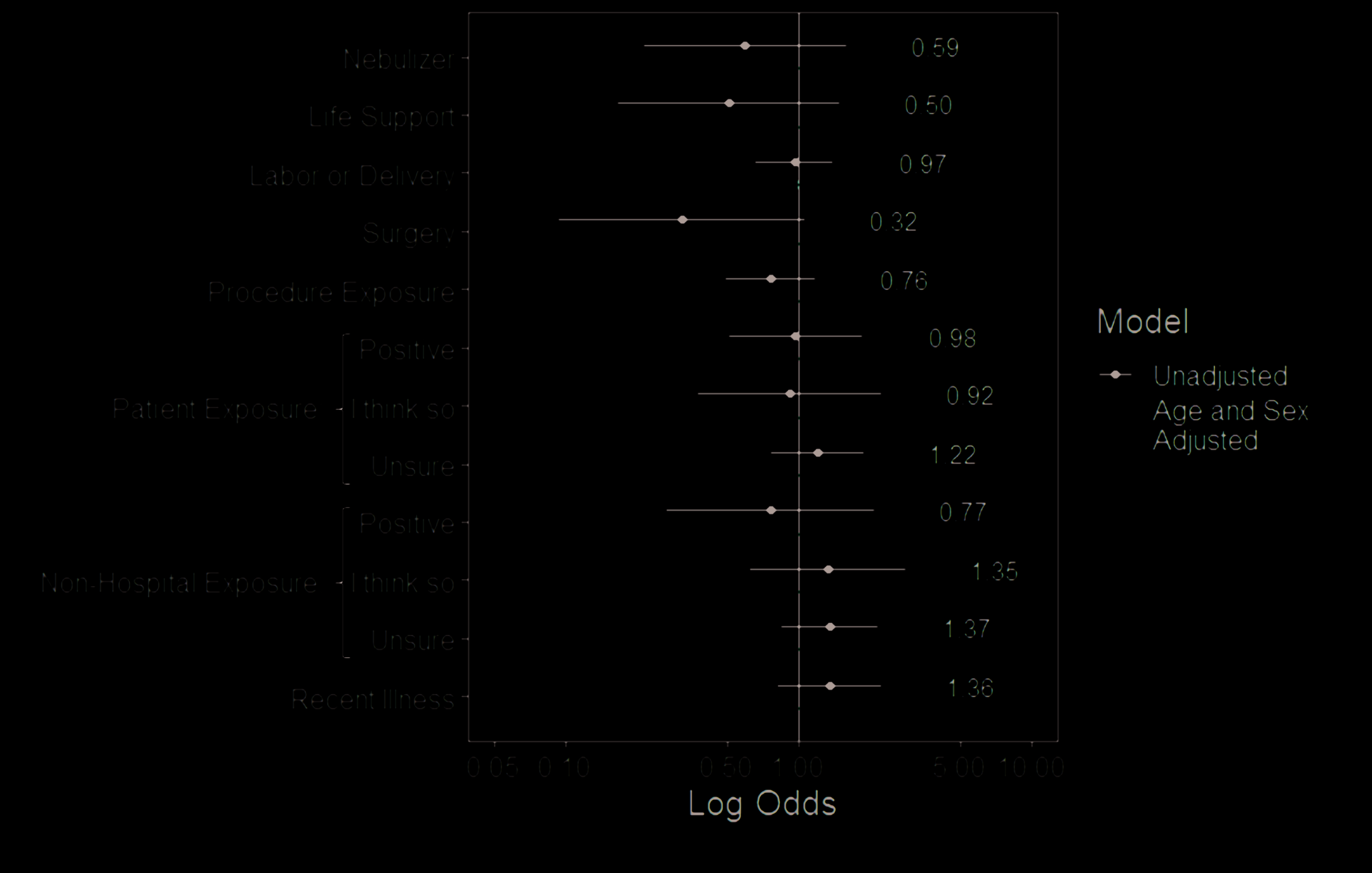

Supplement: S5 Fig — (TIF) [file pgph.0000549.s007.tif]
