# Supplementary material for: Nigeria healthcare worker SARS-CoV-2 serology study: Results from a prospective, longitudinal cohort
Source: PLOS Glob Public Health. 2023 Jan 17;3(1):e0000549. doi: 10.1371/journal.pgph.0000549 (PMC10022168; doi:10.1371/journal.pgph.0000549)
Supplement: Attachment — Submitted filename: Seroprevalence PLoS GPH Response_21.04.22.docx [file pgph.0000549.s008.docx]

**RESPONSE TO COMMENTS**

**“NIGERIA HEALTHCARE WORKER SARS-COV-2 SEROLOGY STUDY: RESULTS FROM A PROSPECTIVE, LONGITUDINAL COHORT”**

**PGPH-D-21-01149**

**EDITORS’ COMMENTS**

**1. Elaborating more the baseline of the study:**

**a. methods - a dried blood spot IgG assay to determine the presence of SARS-CoV-2 specific IgG – not only refer to the literature but also describe.**

**b. baseline seropositive individuals who reported COVID-19.**

**c. how many reported COVID-19 in follow up (3- and 6-months).**

Response:

For 1a, we have adapted the text to more thoroughly describe how the assay was validated and how the sample analyses were conducted, including whether the method detects IgG to previous coronavirus infections and information about the use of seronegative blood samples that were collected prior to the COVID-19 pandemic. We have previously included discussion of the limitations of the validation in the Discussion section.

For 1b, out of 150 participants who were seropositive at baseline, 16 underwent diagnostic testing for COVID, and all (16/16) reported receiving negative test results. Thus, 0% were reported as positive. We did not include a time window about baseline symptoms and testing preceding the seroprevalence study. The testing question includes language "excludes testing for the current study", and we did not conduct antigen or PCR diagnostic tests.

For 1c, the number of individuals who tested positive and negative for COVID is outlined below. These results have a relatively large number of respondents who were either awaiting results or did not know the results, and thus, this table is not particularly informative. Given the reviewer’s later recommendation to streamline the tables and figures, we do not propose including these results.

| **Time Point** | **Got tested for COVID *(excluding test done for study)*** | **Results** | | |
| --- | --- | --- | --- | --- |
|  |  | **Positive** | **Negative** | **Awaiting results/Don’t know** |
| **Baseline** | 61 | 4 | 53 | 4 |
| **Month 3 follow up** | 5 | 1 | 3 | 1 |
| **Month 6 follow up** | 24 | 8 | 13 | 3 |

Missing information on COVID testing: Baseline: 2, Month 3: 16, and Month 6: 55.

*Changes to manuscript (Methods):*

*“Samples were analyzed using a validated dried blood spot assay specific to SARS-CoV-2 (McDade et al. 2020). This method was adapted from a quantitative ELISA that was granted Emergency Use Authorization from the US Food and Drug Administration to measure SARS-CoV-2 antibodies in serum and that was determined to not cross-react with other common coronavirus strains (Amanat et al. 2020; McDade et al. 2020). Briefly, the ELISA method was validated using an antibody (CR3022) with defined affinity to the receptor binding domain of the spike protein (McDade et al. 2020). To determine seropositivity for the presence of SARS-CoV-2 antibodies, the cut-off was set at the optical density value produced by the 0.39 μg/ml CR3022 calibrator. This low seropositive cut-off is greater than 3 standard deviations above optical density values for 23 known negative samples collected in 2018, and it is well above the assay’s lower limit of detection. The method shows strong agreement between matched dried blood spot and serum samples from 17 individuals (R^2^=0.9945).”*

**d. COVID vaccination during study period.**

Response: The study period (July 2020 to December 2020) preceded the start of COVID-19 vaccinations in Nigeria, which began on March 5, 2021. (https://covid19.who.int/region/afro/country/ng)

*Changes to manuscript:*

*“Seroprevalence substantially increased over the 6-month follow-up period (70%), a period that included the second wave of cases and preceded the start of COVID-19 vaccinations in Nigeria (March 5, 2021).”*

**e. SARS-CoV-2 variants circulating during study period.**

Response: Coordination of genomic surveillance among the 3 laboratories with genomic surveillance capacity in Nigeria (Nigerian Centre for Disease Control, the Nigeria Institute for Medical Research, and the Africa Centre for Excellence in Genomics) did not commence until February 2021. (https://covid19.ncdc.gov.ng/media/files/STATEMENT_ON_VARIANTS_OF_SARS-COV-2_IN_NIGERIA_ZyRAI48.pdf) Thus, data on variants of concern, variants under monitoring, or variants being monitored are limited during the study period.

Nevertheless, data published in February 2022 from Oyo state demonstrate Eta variant (B1.525) circulation between July 2020 and December 2021 among a sample of 378 SARS CoV-2 isolates (mean age=42 years, 44% female; Ozer E, et al. Nature Communication, 2022), which was also reported by the Nigerian Centre for Disease Control. Phylogenetic analyses demonstrate two primary lineages circulating in 2021: B.1.1.7 (Alpha) and B.1.525 (Eta). The delta variant that began circulating widely in Nigeria in June 2021 was also noted to be a globally uncommon sub-variant AY.36, which accounted for more than 80% of delta cases in Nigeria but less than 0.5% of delta cases in other contexts. Results from other states had a generally similar distribution, albeit with a lower prevalence of Eta variant.

*Changes to manuscript (Discussion section):*

*“While genomic surveillance was limited in Nigeria during the study period, results from phylogenetic analyses suggest that variants B.1.1.7 (Alpha) and B.1.525 (Eta) were the most commonly circulating variants introduced in fall 2020.”*

**2. Reformulating of the results and reorganize the graphical presentation of the results:**

**a. consider simplifying and/or combining figures and tables.**

Response: Figure 2 has been simplified. Supplemental Figure 1 has been changed to respond to the Editor’s next comment (#2b), and Supplemental Tables 2 and 3 have been removed.

*Changes to manuscript: As above.*

**b. Explain age differences among (those who) developed symptomatic COVID compared to asymptomatic COVID.**

Response: Data from the WHO UNITY systematic review of seroprevalence studies suggest no difference in rates of asymptomatic prevalence among older versus younger individuals (51.1% in ages 60+ years to 71.0% in ages 20-29 years (Kruskal-Wallis (KW) H-test p = 0.45). Results from the current study do not show any age-related differences. We report symptom-related results for individuals <40 years and ≥ 40 years in the new Supplemental Table 1.

| Baseline Characteristic | Total respondents  N (525) | Age <40 years  (N=278) | Age ≥ 40 years  (N=247) | *P-*value |
| --- | --- | --- | --- | --- |
| Any symptoms, No. (%) | 509 | 32 (11.8) | 32 (13.4) | 0.58 |
| Fever, No. (%) | 64 | 20 (62.5) | 22 (68.8) | 0.60 |
| Shortness of breath, No. (%) | 63 | 1 (3.2) | 0 (0.0) | 0.31 |
| Chest congestion, No. (%) | 63 | 1 (3.2) | 0 (0.0) | 0.31 |
| Chest tightness, No. (%) | 63 | 0 (0.0) | 2 (6.3) | 0.16 |
| Dry cough, No. (%) | 61 | 1 (3.4) | 1 (3.1) | 0.94 |
| Wet/loose cough, No. (%) | 61 | 1 (3.4) | 3 (9.4) | 0.35 |
| Body aches or pains, No. (%) | 61 | 16 (55.2) | 24 (75.0) | 0.10 |
| Chills or shivering, No. (%) | 62 | 8 (26.7) | 4 (12.5) | 0.16 |
| Sore throat, No. (%) | 62 | 1 (3.3) | 2 (6.3) | 0.59 |
| Congested or runny nose, No. (%) | 62 | 6 (20.0) | 5 (15.6) | 0.65 |
| Diarrhea, No. (%) | 62 | 3 (10.0) | 1 (3.1) | 0.27 |
| Weak or tired, No. (%) | 61 | 20 (66.7) | 16 (51.6) | 0.23 |
| Loss of smell, No. (%) | 61 | 0 (0.0) | 1 (3.1) | 0.34 |
| Loss of taste, No. (%) | 62 | 4 (13.3) | 2 (6.3) | 0.35 |
| Loss of appetite, No. (%) | 62 | 11 (36.7) | 9 (28.1) | 0.47 |
| Vomiting, No. (%) | 61 | 1 (3.3) | 1 (3.2) | 0.98 |
| Nausea, No. (%) | 61 | 1 (3.4) | 4 (12.5) | 0.20 |
| Headache, No. (%) | 62 | 25 (83.3) | 19 (59.4) | 0.04 |
| Abdominal pain, No. (%) | 62 | 4 (13.3) | 2 (6.3) | 0.35 |

*Excluded: 16 participants were missing information on questions about symptoms. 15 participants believed they had COVID-19 because they believed they has illness but either tested negative/did not test and 1 was truly missing information*.

*Changes to manuscript: Supplemental Table 1 updated.*

**c. Elaborate SARS-CoV-2 variants differences among developed symptomatic COVID compared to asymptomatic COVID.**

Response: Building upon comments outlined in response to Editor Comment 1e, the dominant variants in Nigeria during at least some part of the study period were B.1.1.7 (Alpha) and B.1.525 (Eta). However, the current study did not collect data on variants among individuals who had COVID-19, regardless of symptoms, and thus this question is beyond the scope of the current study.

*Changes to manuscript: None.*

**3. Rewriting the discussion - essentially modify this section with a special attention to the recently published works on SARS-CoV-2 seroprevalence in Africa using the sources suggested by the reviewer.**

Response: The manuscript has been updated to include the recommended data and references, including in the Introduction and Discussion sections.

*Changes to manuscript:*

Introduction section:

*“National seroprevalence has been estimated to be 78·9% (95% CI: 77·7, 80·0) based on data collected in Q3 2021.”*

Discussion section:

*“A 2021 scoping review of seroprevalence studies among healthcare workers in 11 African countries, which overlapped with the current study period (April 2020 to December 2020), demonstrated a seroprevalence range of 0% to 45·1%.”*

*“Results from the World Health Organization UNITY Studies Collaborative Group, which aims to synthesize data from seroprevalence studies across Africa (n=151 studies overall; n=7 studies from Nigeria), have shown substantial geographic and temporal variation. SARS-CoV-2 seroprevalence estimates increased from 3.0% (95% CI: 1.0, 9.2%) in Q2 2020 to 65.1% (95% CI: 56.3, 73.0%) in Q3 2021 among all studies. During the current study period, seropositivity reported in regional or subnational Nigerian studies ranged from 9.3% (95% CI: 6.9, 11.6%) in Gombe State to 25.4% (95% CI: 19.3-32.3%) in Niger State. In Nigeria’s only national seroprevalence study conducted in Q3 2021, seropositivity was 78.9% (95% CI: 77.7, 80.0).”*

**JOURNAL REQUIREMENTS:**

**1. Please provide additional details regarding participant consent.**

Response: We outline the collection of informed consent from adult participants in the Methods section.

*Changes to manuscript: None*

**2. Please update the completed 'Competing Interests' statement. Please declare all competing interests beginning with the statement “I have read the journal's policy and the authors of this manuscript have the following competing interests:”**

Response: The Competing Interests statement has been updated.

*Changes to manuscript: Statement updated.*

**3. Please remove the statement “The funders had no role in the design, conduct of the study; collection, management, analysis, and interpretation of the data; preparation, review, or approval of the manuscript; or decision to submit the manuscript for publication." in your 'Competing Interests' statement.**

Response: The statement has been removed.

*Changes to manuscript: Statement removed.*

**4. In the online submission form, you indicated that your data will be submitted to a repository upon acceptance. We strongly recommend all authors deposit their data before acceptance, as the process can be lengthy and hold up publication timelines. Please note that, though access restrictions are acceptable now, your entire data will need to be made freely accessible if your manuscript is accepted for publication. This policy applies to all data except where public deposition would breach compliance with the protocol approved by your research ethics board. If you are unable to adhere to our open data policy, please kindly revise your statement to explain your reasoning and we will seek the editor's input on an exemption. Please be assured that, once you have provided your new statement, the assessment of your exemption will not hold up the peer review process.**

Response: We have updated the Data Sharing statement to align with sharing at the time of manuscript acceptance rather than manuscript publication.

*Change to manuscript:*

*“Individual participant data on the results reported in this article (texts, tables, figures, and supplementary material, after de-identification will be made available immediately after acceptance without end date, to anyone who wishes to access the data for any purpose.”*

**5. Have the authors made all data underlying the findings in their manuscript fully available (please refer to the Data Availability Statement at the start of the manuscript PDF file)?**

**The PLOS Data policy requires authors to make all data underlying the findings described in their manuscript fully available without restriction, with rare exception. The data should be provided as part of the manuscript or its supporting information, or deposited to a public repository. For example, in addition to summary statistics, the data points behind means, medians and variance measures should be available. If there are restrictions on publicly sharing data—e.g. participant privacy or use of data from a third party—those must be specified.**

Response: We have updated the Data Sharing statement.

*Change to manuscript:*

*“Individual participant data on the results reported in this article (texts, tables, figures, and supplementary material, after de-identification will be made available immediately after acceptance without end date, to anyone who wishes to access the data for any purpose.”*

**REVIEWERS’ COMMENTS**

**Reviewer #1:**

**1. Serological testing: The authors have used a dried blood spot IgG assay to determine the presence of SARS-CoV-2 specific IgG. They refer (to) a published paper for methods. However, it would be important to have the following information regarding this dried spot assay in this manuscript itself, without just citing a previous published paper as this validity of this assay is a crucial part of this manuscript.**

**1. What was the sensitivity and specificity of this assay compared to standard neutralization assays or any commercial assays? How as it validated?**

Response: See response to Editor’s Comment #1a.

*Change to manuscript: As above.*

**2. Does it detect IgG to previous coronavirus infections? Has this (been) validated in the Nigerian community?**

Response: See response to Editor’s Comment #1a.

*Change to manuscript: As above.*

**3. Was this validated using seronegative blood samples prior to the COVID-19 pandemic? What was the result? On how many samples?**

Response: See response to Editor’s Comment #1a.

*Change to manuscript: As above.*

**2. How many of the baseline seropositive individuals reported COVID-19? Were all these infections asymptomatic?**

Response: See response to Editor’s Comment #1b.

*Change to manuscript: As above.*

**3. In the population followed up at 3 months and 6 months, how many reported COVID-19? Of the individuals who seroconverted between 0 to 3 months and 3 to 6 month time points, how many seroconverted asymptomatically and how many were diagnosed to have COVID?**

Response: See response to Editor’s Comment #1c.

*Change to manuscript: As above.*

**4. Did any individual receive any COVID vaccines at any of the time points?**

Response: See response to Editor’s Comment #1d.

*Change to manuscript: As above.*

**5. What were the SARS-CoV-2 variants circulating during the time of the study? Was there any sequencing done in the country during the time of the study? Because the study was carried out from July 2020 to Jan 2021, there were no variants of concern circulating at that time?**

Response: See response to Editor’s Comment #1e.

*Change to manuscript: As above.*

**6. Were there differences in ages in those who developed symptomatic COVID compared to asymptomatic COVID?**

Response: See response to Editor’s Comment #2b.

*Change to manuscript: As above.*

**7. Based on the study results, the majority of infections in health care workers were asymptomatic? When delta variant was affecting the country, did Nigeria experience more symptomatic infection in HCWs?**

Response: Yes, most participants were asymptomatic, which is consistent with results from the WHO UNITY systematic review, which demonstrated that 67% (95% CI: 47.8-80.9%) of seropositive participants were asymptomatic, based on 14 studies. The study period preceded the discovery of the delta variant into Nigeria, so the second question is beyond the scope of this study. The delta variant was discovered in India in October 2020 (https://www.who.int/en/activities/tracking-SARS-CoV-2-variants/) and was not identified in Nigeria until July 8, 2021 (https://ncdc.gov.ng/news/333/confirmed-case-of-delta-variant-detected-in-nigeria).

*Change to manuscript: None*

**8. Discussion: It would be important to discuss how these findings compare with studies done in a similar time period in health care workers in other countries.**

Response: See response to Editor’s Comment #3.

*Change to manuscript: As above.*

**Reviewer #2:**

**1. It is recommended that authors rewrite their discussion to reference recently published work on SARS-CoV-2 seroprevalence in Africa.**

Response: See response to Editor’s Comment #3.

*Change to manuscript: As above.*

**2. While the authors thoroughly review their results, the abundance of figures and tables making the result section difficult to follow. Authors should consider streamlining and combining figures and tables to better highlight key findings from the study.**

Response: See response to Editor’s Comment #2a.

*Change to manuscript: As above.*

**3. Line 101: authors indicate that “health care workers from 60 primary health care centers and University of Abuja Teaching Hospital” where identified. Yet on line 104 they state that multi-stage probability sampling was performed “among the 243 primary health care centers”. Please clarify this discrepancy.**

Response: The study team used infrastructure from an existing research study within primary healthcare centers to recruit health care workers to participate in the current study. These primary health care centers were recruited based on multi-stage probability sampling, and health care workers were invited to participate in the current study. The University of Abuja Teaching Hospital coordinated this and the existing research study, and health care workers from the University of Abuja Teaching Hospital were similarly invited to participate in the current study. We have clarified this in the Methods section

*Change to manuscript:*

*“The approach to the site-level sampling frame among primary health care centers, which was conducted in 2019 as part of an ongoing research study, has been published.^8^”*

**4. Authors should consider providing the specifications of the serological test used and its performance characteristics. Notably include existing validation data for testing on DBS vs serum. Have authors evaluated possible cross reactivity with other pathogens as reported in the region?**

Response: See response to Editor’s Comment #1a.

*Change to manuscript: As above.*

**5. Line 435: GISAID is a database dedicated to hosting genomics data. It is not clear how this would be relevant to the data from the current study. Authors may instead consider submitting data to Serotracker (https://serotracker.com/en/Data). Further, to comply with PLOS data policy, data and related metadata underlying reported findings should be deposited ahead of publication (https://journals.plos.org/globalpublichealth/s/data-availability).**

Response: The study team will submit de-identified study data to Serotracker. The Data Sharing statement has been updated based on the response to Journal Requirement #4.

*Change to manuscript: As above.*

**6. Line 123: authors should consider providing study ethics approval nb and/or date.**

Response: Information related to ethical approval of the study has been added to the Methods section.

*Change to manuscript:*

*“The clinical and data coordinating center was based at the University of Abuja Teaching Hospital, which provided ethical review and approval of the study procedures, along with the Institutional Review Board of Northwestern University (STU00212900).”*

**7. Line 124: REDCap was used to obtain informed consent: can authors clarify how that was done? was it an electronic signature?**

Response: Consent was obtained through an electronic signature.

Change to manuscript:

*“Trained study staff conducted interviewer-administered surveys after informed consent was received through an electronic signature.”*

**8. Line 128 to 131: content repeat line 95-97. Suggest deleting one of these instances to avoid redundancy.**

Response: We agree with this suggestion.

*Change to manuscript: Text in lines 95-97 removed.*

**9. Line 212 to 213: supplemental tables 3 and 4 are cited but do not seem to be included in the manuscript.**

Response: We have removed Supplemental Table 3 based on previous suggestions and have ensured that Supplemental Table 4 (now listed as Supplemental Table 2 because of the removal of the previous Supplemental Table 2 and 3) is included in the submission.

*Change to manuscript: Supplemental Table 3 deleted. Text updated.*

**10. Line 279 to 293: Authors should consider updating these paragraphs to reflect currently available data about SARS-CoV-2 seroprevalence in Africa.**

Response: See response to Editor’s Comment #3.

*Change to manuscript: As above.*
