# Supplementary material for: Nigeria healthcare worker SARS-CoV-2 serology study: Results from a prospective, longitudinal cohort
Source: PLOS Glob Public Health. 2023 Jan 17;3(1):e0000549. doi: 10.1371/journal.pgph.0000549 (PMC10022168; doi:10.1371/journal.pgph.0000549)
Supplement: Attachment — Submitted filename: Seroprevalence PLoS GPH_Response to Reviewers_15.10.22.docx [file pgph.0000549.s009.docx]

**RESPONSE TO COMMENTS**

**“NIGERIA HEALTHCARE WORKER SARS-COV-2 SEROLOGY STUDY: RESULTS FROM A PROSPECTIVE, LONGITUDINAL COHORT”**

**PGPH-D-21-01149**

We want to appreciate the Editor and the reviewers for finding the time to review our manuscript. We have responded to the Editor’s and Reviewer 3 comments below.

The comments are in bold while the responses are just below.

**Editor’s Comments and Responses**

**1. Please provide additional details regarding participant consent. In the ethics statement in the Methods and online submission information, please ensure that you have specified whether consent was written or verbal/oral. If consent was verbal/oral, please specify: 1) whether the ethics committee approved the verbal/oral consent procedure, 2) why written consent could not be obtained, and 3) how verbal/oral consent was recorded. If your study included minors, please state whether you obtained consent from parents or guardians in these cases. If the need for consent was waived by the ethics committee, please include this information.**

REDCap was used to obtain informed consent from all participants and this was approved of by the University of Abuja Human Ethics Committee. This has been added in the ‘Materials and Methods’ lines 145-147.

**Please review your reference list to ensure that it is complete and correct. If you have cited papers that have been retracted, please include the rationale for doing so in the manuscript text, or remove these references and replace them with relevant current references. Any changes to the reference list should be mentioned in the rebuttal letter that accompanies your revised manuscript. If you need to cite a retracted article, indicate the article’s retracted status in the References list and also include a citation and full reference for the retraction notice.**

The references have been reviewed and they are fine.

**This is a very well written manuscript on a relevant subject, many different results besides the prevalence itself are interesting, such as the absence of symptoms in most, indicating that infection was much more frequent than possibly reported by countries. A sentence on this would be nice in the discussion. While revising your text as recommended by reviewer 3, please also include a sentence on possible selection bias from the recruitment method (active response of an invitation, HCW with more concern would have possibly responded more positively). Congratulations on your work.**

We want to thank the Editor for acknowledging the importance of this manuscript. To address the comments, we have added the sentence below to the discussion section (lines 372-374):

The high prevalence of seropositivity in this study despite the the absence of symptoms in most participants indicate that infection was much more frequent than possibly reported by many countries.

**In table -3, it is unclear what models 1, 2 & 3 mean. It would be better to describe in methods section and to written below table-3.**

In the statistical portion of the methods section, it was previously stated that unadjusted and adjusted multivariate logistic regression models were created to evaluate association between baseline characteristics and IgG seropositivity to SARS-CoV-2 at baseline and each follow-up. To clarify this further, I have copied sentences in lines 192-200 of the manuscript below to clarify this further:

‘Unadjusted and adjusted multivariate logistic regression models were created to evaluate association between baseline characteristics and IgG seropositivity to SARS-CoV-2 at baseline and each follow-up. Sociodemographic characteristics, including area council of participants’ work (work area council), and medical history, including COVID-19 testing and diagnosis, were assessed as risk factors associated with SARS-CoV-2 seropositivity. Multivariate models were adjusted for age, sex, BMI, occupation, work area council, and history of immunocompromised state based on a previous report^9^, as well as adjustment for within within-work location (i.e., primary health care center or hospital) clustering. Participants’ home area council was considered in these models but was collinear with work council area.’

For clarity, I have added the sentence below under table 3

‘Model 1: Unadjusted results. Model 2(*): Adjusted for age and sex. Model 3 (^†^): Adjusted for age, sex, occupation, work council area, and history of immunocompromised state.’
